# Drought characteristics and its elevation dependence in the Qinghai–Tibet plateau during the last half-century

**DOI:** 10.1038/s41598-020-71295-1

**Published:** 2020-08-31

**Authors:** Wei Feng, Hongwei Lu, Tianci Yao, Qing Yu

**Affiliations:** 1 Key Laboratory of Water Cycle and Related Land Surface Processes, Institute of Geographic Sciences and Natural Resources Research, Chinese Academy of Sciences, Beijing, 100101 China; 2 University of Chinese Academy of Sciences, Beijing, 100049 China

**Keywords:** Climate sciences, Hydrology, Natural hazards

## Abstract

Associated with global warming, drought has destructive influences on agriculture and ecosystems, especially in the fragile Qinghai–Tibet Plateau (QTP). This study investigated spatial–temporal patterns of meteorological drought in the QTP and its surrounding areas and made an attempt to explore the relationship between drought conditions and elevation. Robust monitoring data from 274 meteorological stations during 1970–2017 were analyzed using the Sen’s slope method, Mann–Kendall trend test and rescaled range analysis. Results revealed that under the wetting trend in the QTP, Standardized Precipitation Evapotranspiration Index (SPEI) increased of maximum 0.012/year in spring. Moreover, severe drought frequency in winter and future drought risk in summer also showed an increasing trend. Wetter trends were positively correlated with elevation, with a key point being 4,000 m where the change trend above 4,000 m was about 6.3 times of that below 4,000 m in study area. The difference of drought severities between SPEI in the QTP and its surrounding areas has increased from − 0.19 in 1970 to 0.38 in 2017 and kept growing in future.

## Introduction

Drought is one of the most widespread and costly natural disasters^1^, which can endanger the production of agriculture and animal husbandry, worsen the ecological environment, and even expose human to the risk of disease^2,3^. Previous studies have suggested that under global warming, the percentage of dry areas in the world has increased by approximately 1.74% per decade during 1950–2008^4,5^. With an average elevation above 4,000 m and an area of 200,000 square kilometers, the Qinghai–Tibet Plateau (QTP) is the source of major rivers in Asia^6–8^. It is extremely vulnerable to global change, and easily suffers from drought. The drought herein will have profound impacts on the neighboring regions. Therefore, a comprehensive understanding of drought characteristics in the QTP is of great importance.

Many studies on spatiotemporal characteristics of drought in the QTP and its surrounding areas have been conducted, mainly concluded that the QTP has become warmer and wetter in the past decades, especially in the vast northwestern QTP^5–7,9–13^. Additionally, Gao et al.^8^ analyzed the aridity changes using P/PET ratio in recent 30 years based on 83 stations, and found that the eastern QTP was becoming drier and the aridity change pattern was significantly correlated with precipitation, sunshine duration and diurnal temperature range. Liang et al.^14^ investigated 74 stations in the QTP during 1980–2014 and found that the drought pattern exhibited obvious inter-decadal variation and severe drought mainly occurred before the 1990s. Yang et al.^15^ forecasted an increasing drought trend in southwest China (including Yunnan Province) but an increasing wetting trend for the QTP based on simulation of Global Climate Models (GCM) taken from the Coupled Model Intercomparison Project Phase 5 (CMIP5) framework. Other studies have addressed the seasonal drought evolution in the QTP. Some concluded that the drought mainly decreased in spring, and a slight drying trend could be traced in winter^16,17^; in autumn, extreme drought frequency increased in the eastern QTP but decreased in the northern region. Wang et al.^18^ used the self-calibrating Palmer Drought Severity Index (scPDSI) to investigate the drought variation between 1961–2009 and revealed that the southern QTP experienced a significant wetting trend although the northern QTP became significantly drier, particularly in spring and autumn. Apparently, different opinions exist in seasonal drought variations in the QTP and further investigations are thus desired.

Understanding the climatic trends in highland regions is important and necessary for supporting global change studies because of their sensitivity and vulnerability to climate change^19^. Due to the drastic elevation changes, the QTP is a favorable place to explore the relationship between climate change and elevation. According to previous studies, mountainous areas are more sensitive to climate change compared to low-altitude areas at the same latitude^20–22^. In recent years, many scientists have focused on elevation-related climate change researches, confirming the evidences of the elevation-dependent warming^21,23^. Some have revealed that the warming trend displayed a slight decrease with elevation over 4,000 m^24^, Zhang et al.^25^ found smaller changes of PET in high-elevation areas at both annual and seasonal scales. Li et al.^26^ concluded an increasing tendency of the precipitation with increasing elevation in summer. However, knowledge on elevation dependence of drying or wetting trends over the QTP is not well understood, particularly under low monitoring data availability and complex terrain conditions (low remote-sensing applicability).

Investigating drought trends in mountainous regions at different time scales along the elevation gradient is of great significance for thoroughly exploring drought phenomenon. In this study, monitoring data from 274 meteorological stations in the past 48 years were used to analyze wet and dry conditions evolutions over the QTP and its surrounding areas. The main objectives of this study were to: (1) assess the spatial distribution and temporal variation of drought, particularly severe drought in the QTP during 1970 to 2017; (2) explore how drought changes with elevation in the QTP and their possible causes; (3) discuss the persistence of drought trends.

## Materials and methods

### Study area and data

**Figure 1:**
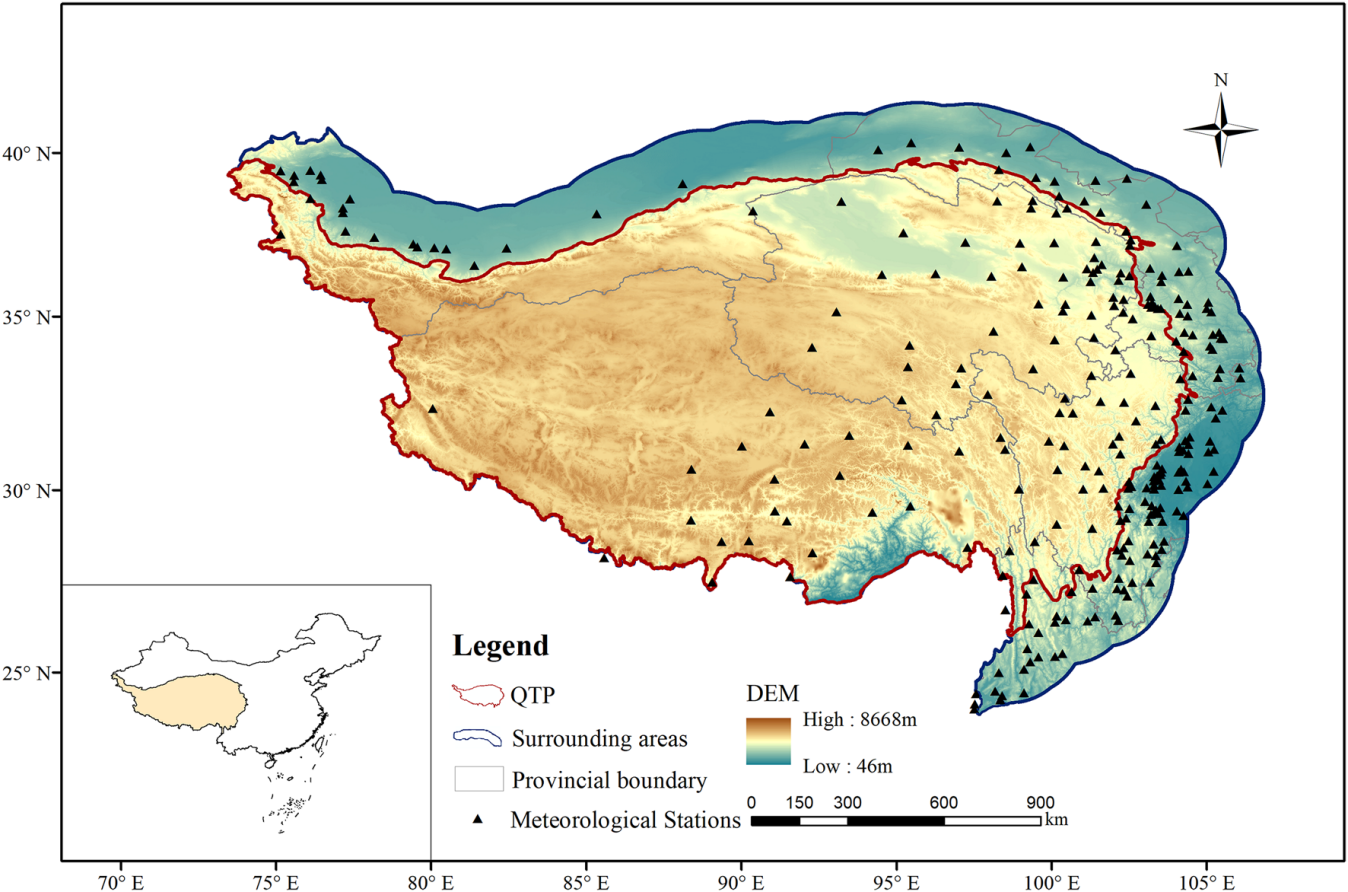
Study region and location of meteorological stations. The map was created using ArcMap 10.2, URL: https://www.esrichina-bj.cn/softwareproduct/ArcGIS/.

The study area locates in southwest China (24.0–40.3°N, 75.1–106.1°E) (Fig. 1). In order to explore the spatial–temporal pattern of drought in the QTP, particularly relationships between drought and elevation, a 200 km buffer zone based on the QTP boundary within the Chinese border was established by using the ArcGIS 10.2, namely “the surrounding areas”. The study area is thus composed of two parts, i.e. QTP and the surrounding areas. It includes Qinghai Province, Tibet Autonomous Region, part of Gansu Province, northern Yunnan Province, western Sichuan Province, part of Ningxia Hui Autonomous Region and southern Xinjiang Uygur Autonomous Region (Fig. 1). The average annual temperature of the study region reduced from 22 °C in the southeast to below − 7 °C in the northwest. As the warm and humid air mass moving from the Indian Ocean is blocked by huge mountains, average annual precipitation has also reduced from 2,597 mm to less than 1.9 mm from southeast to northwest. The QTP is the origin of many rivers in Asia including the Yarlung Zangbo River, Nu River, Yangtze River, Yellow River and Lancang River. It also comprises a series of high mountains such us the Kunlun, Qilian, Tanggula and Hengduan mountains. Because of its special geographic location and large-scale topography, the QTP has a strong impact on both regional and global climates.

The meteorological data (i.e. daily precipitation and temperature) covering 274 stations (Fig. 1) during 1970–2017 were obtained from the Data Center for Resources and Environmental Science, Chinese Academy of Sciences (https://www.resdc.cn/). The dataset has been widely used in many studies^27–30^. 115 of them locate in the QTP and 159 of them are distributed in the buffer zone. The originally observed data were processed through standard quality control by the Data Center.

### Methods

#### Standardized precipitation evapotranspiration index

SPEI as an improved drought index of SPI was first proposed by Vicente-Serrano et al.^31^. It has many advantages and was widely used in many studies^17,32,33^. Compared to SPI, it adds temperature upon precipitation and can reveal the effects of global warming on drought^34^. Potential evapotranspiration (PET) is a key part of the SPEI. Different methods have been proposed to estimate PET over the past decades. Some of them are based on physical mechanism, such as the FAO-56 Penman–Monteith method (PM), and the others arose from empirical relationships (e.g. Thornthwaite method^35^, TH) that need less parameters. The previous studies show that aerodynamic factors often have impacts in spring and winter of northern China, but the overall estimation of PET (in both temporal evolution and spatial distribution) from two methods are very comparable^34,36^. Similar conclusions can also be found in Vicente-Serrano et al.^31^ and Mavromatis^37^. Therefore, we adopted the TH method to calculate PET and SPEI considering data availability and natural features of QTP.

In this study, SPEI-annual and SPEI-seasonal were computed by using the SPEI package in the R software^38^. The SPEI-annual and SPEI-seasonal were calculated from accumulation of climatic water balance during a 12-month period from the month to the preceding 12 months and a 3-month period from the month to the preceding 3 months, respectively. Among them, we identified specific SPEI values to present annual and seasonal conditions, i.e. SPEI-annual (the SPEI-12 of December) and SPEI-seasonal (the SPEI-3 of May, August, November and the next February for SPEI-spring, SPEI-summer, SPEI-autumn and SPEI-winter, respectively), linking to an estimation of meteorological drought. The detailed calculation steps of SPEI can be found in Vicente-Serrano et al.^31^. Table 1 shows the tentative range of SPEI and drought grade classification criteria.

**Table 1:** Categories of drought grade based on SPEI^1^.

Categories	SPEI values
No drought	− 0.5 < SPEI
Mild drought	− 1.0 < SPEI ≤ − 0.5
Moderate drought	− 1.5 < SPEI ≤ − 1.0
Severe drought	− 2.0 < SPEI ≤ − 1.5
Extreme drought	SPEI ≤ − 2.0

#### Sen’s slope

When using Mann–Kendall trend test (MK-test) to detect a changing trend of time series, Sen's slope is usually employed to estimate the magnitude of the trend as follows^39^:

1 $$f(t) = Mt + C$$

where *f*(*t*) is the function of the linear trend, *M* is the slope and *C* is the constant of the equation.

The formula of the trend’s magnitude estimation is:

2 $$Q = median\frac{{X_{i} - X_{j} }}{{t_{i} - t_{j} }}$$

where *x*_i_ and *x*_j_ are the data values at times *t*_*i*_ and *t*_*j*_ (i > j), respectively.

#### Mann–Kendall test

The MK-test has been widely used in detecting the significance of Sen’s slope of meteorological factors. In this study, the null hypothesis (H_0_) is the SPEI series (*x*_*1*_, *x*_*2*_, *x*_*3*_, …), which is an independent and uniformly distributed random variable with *n* data points, the alternative hypothesis (H_1_) is a bilateral test, for all *k*, *j* ≤ *n* and *k* ≠ *j, x*_*k*_ and *x*_*j*_ are distributed differently. The test statistical variable *S* is computed as follows:

3 $$S = \sum\limits_{k = 1}^{n - 1} {\sum\limits_{j = k + 1}^{n} {Sgn(x_{j} - x_{k} )} }$$

where *Sgn* (*x*) represents the sign function, which is computed as follows:

4 $$Sgn(x_{j} - x_{k} ) = \left\{ {\begin{array}{*{20}c} { + 1} \\ 0 \\ { - 1} \\ \end{array} \, \begin{array}{*{20}c} {x_{j} - x_{k} > 0} \\ {x_{j} - x_{k} = 0} \\ {x_{j} - x_{k} < 0} \\ \end{array} } \right\}$$

*S* is distributed normally with a mean value of zero, and the variance can be expressed as:

5 $$V{\text{ar}} = n(n - 1)(2n + 5)/18$$

if* n* exceeded 10, the standard test statistical variable *Z* is computed as follows:

6 $$Z = \left\{ {\begin{array}{*{20}c} {\frac{S - 1}{{\sqrt {Var(S)} }}} \\ 0 \\ {\frac{S + 1}{{\sqrt {Var(S)} }}} \\ \end{array} } \right.\begin{array}{*{20}c} {S > 0} \\ {S = 0} \\ {S < 0} \\ \end{array}$$

In the bilateral test, if |*Z*| is ≥ Z_1−(*p*/2)_ at the *p* significance level, the null hypothesis (H_0_) is rejected, i.e., under the given confidence level of *p*, the time series show an upward (*Z* > 0) or downward trend (*Z* < 0). Specifically, *|Z|*≥ 1.28, 1.96 or 2.32 represent the trend of the time series passed the 90%, 95% or 99% confidence levels, respectively.

#### Rescaled range analysis

The Hurst index is used to predict the persistence of the time series. It can be computed by the method of rescaled range analysis (R/S)^25,26^. The following are the calculation steps:

Firstly, divide the SPEI series (U) with length A into [*A/B*] subsequences *u*_*i*_ (*i* = 2, 3, … [*A/B*]) with length B. The subsequences’ extents are computed using the following formula:

7 $$R_{u} = maxZ_{u} - minZ_{u}$$

where *Z*_*u*_ is the sequence cumulative bias of the subsequence of *u*_*i*_.

Secondly, calculate Hurst's empirical formula (*R*_*N*_/*S*_*N*_ = *ωN*^H^) for logarithmic processing as follows:

8 $$log\left( {R_{N} /S_{N} } \right) = HlogN + log\omega$$

where *S*_*N*_ is the standard bias of subsequence *u*_*i*_, *R*_*N*_/*S*_*N*_ is each subsequence’s rescaled range, *H* is the Hurst index, and *ω *is a constant.

*H* ranges from 0 to 1 and can be categorized into three different intervals^40,41^. If 0 < *H* < 0.5, the trend of SPEI series in the future will reverse the current trend; if 0.5 < *H* < 1, the SPEI series is likely to keep the current trend; and *H* = 0.5 indicates the SPEI series will exhibit a random trend in the future.

## Results

### Trend analysis of SPEI series

**Figure 2:**
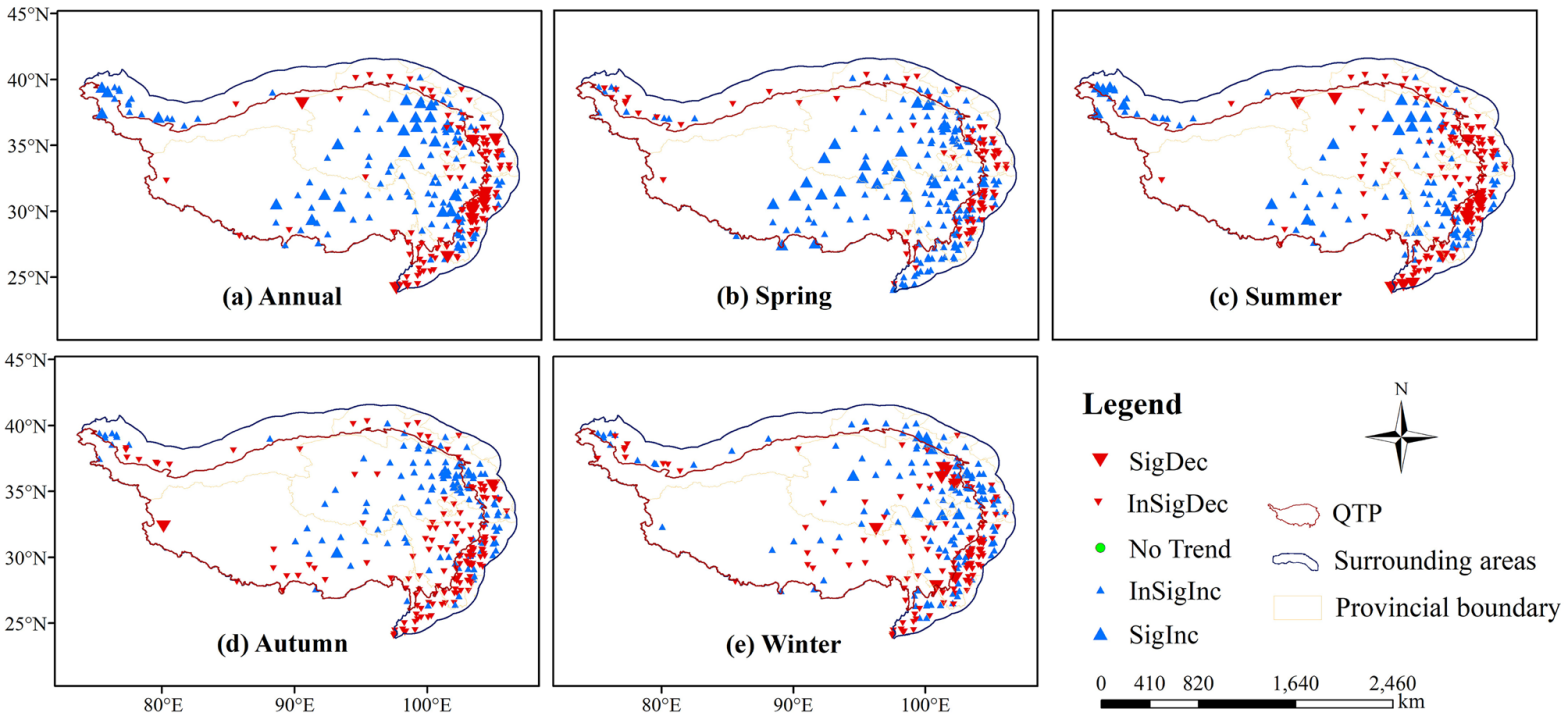
Annual and seasonal distributions of Z values at 274 meteorological stations: (**a**) Annual, (**b**) Spring, (**c**) Summer, (**d**) Autumn, (**e**) Winter. Blue, red triangle and green circle represent wetting, drying and no trends, respectively. The larger triangle indicates significant trends at 95% confidence level. The map was created using ArcMap 10.2, URL: https://www.esrichina-bj.cn/softwareproduct/ArcGIS/, the final figure was generated using Photoshop CC2018, URL: https://onesoftwares.net/.

Figure 2 showed annual and seasonal distributions of temporal trends characterized by Z values at 274 meteorological stations. Annually, the SPEI-annual exhibited an increasing trend in more than 70% of the stations across the QTP (Fig. 2a), illustrating that most of the QTP were getting wetter with a mean rate of 0.0073/year (Table 2). While the surrounding areas were getting drier at the rate of − 0.0033/year with SPEI-annual decreasing in 64.8% stations. A total of 8 stations got drier significantly, mainly distributed in Gansu and Sichuan Provinces, but most stations in Yunnan Province showed slight drying trends.

**Table 2:** Annual and seasonal SPEI trends in the QTP and its surrounding areas.

Area	Annual	Spring	Summer	Autumn	Winter
QTP	0.0073	0.0114*	0.0023	0.0027	0.0030
Surrounding areas	− 0.0033	0.0002	− 0.0036	− 0.0045	0.0020

unit:/year. * indicates trends significant at *p* < 0.05.

Four seasons also experienced wetter trends in the QTP with the most significant trend of 0.0114/year in spring (Table 2, *p* < 0.05), when 90 stations showed an increasing trend and 21 of them were significant. While 15 drying stations were concentrated in a small part of Gansu and Sichuan Provinces (Fig. 2b). In summer, autumn and winter, more than 52% of the QTP stations got an increasing SPEI-seasonal, but approximately 65% of the surrounding stations showed decreasing SPEI-seasonal, indicating a drying trend contrary to the QTP (Fig. 2c–e).

**Figure 3:**
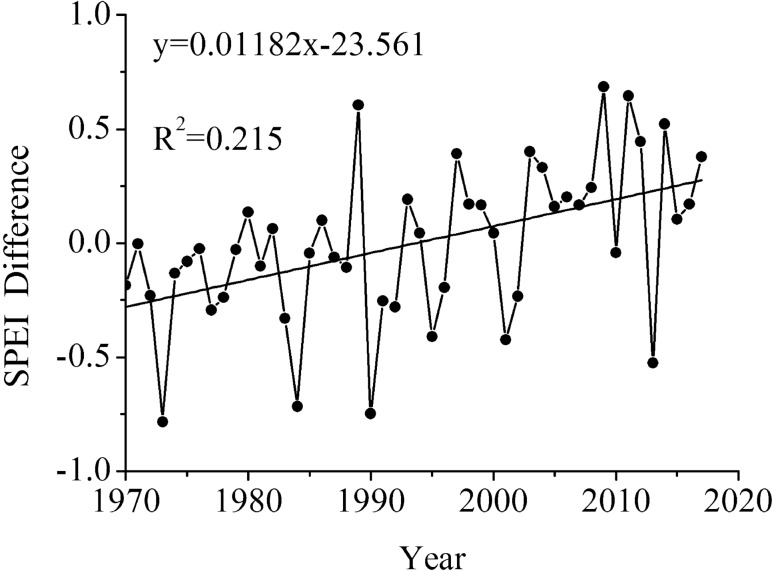
The difference of annual SPEI in the QTP and its surrounding areas.

Apparently, the QTP was getting wetter particularly in spring during the past half century, while the surrounding areas (southeast part, in special) got significantly drier. The QTP had ever been drier than the surrounding areas in the early period but became wetter after 1994, and the difference kept growing up from then on (Fig. 3).

### Temporal variation of severe drought

**Figure 4:**
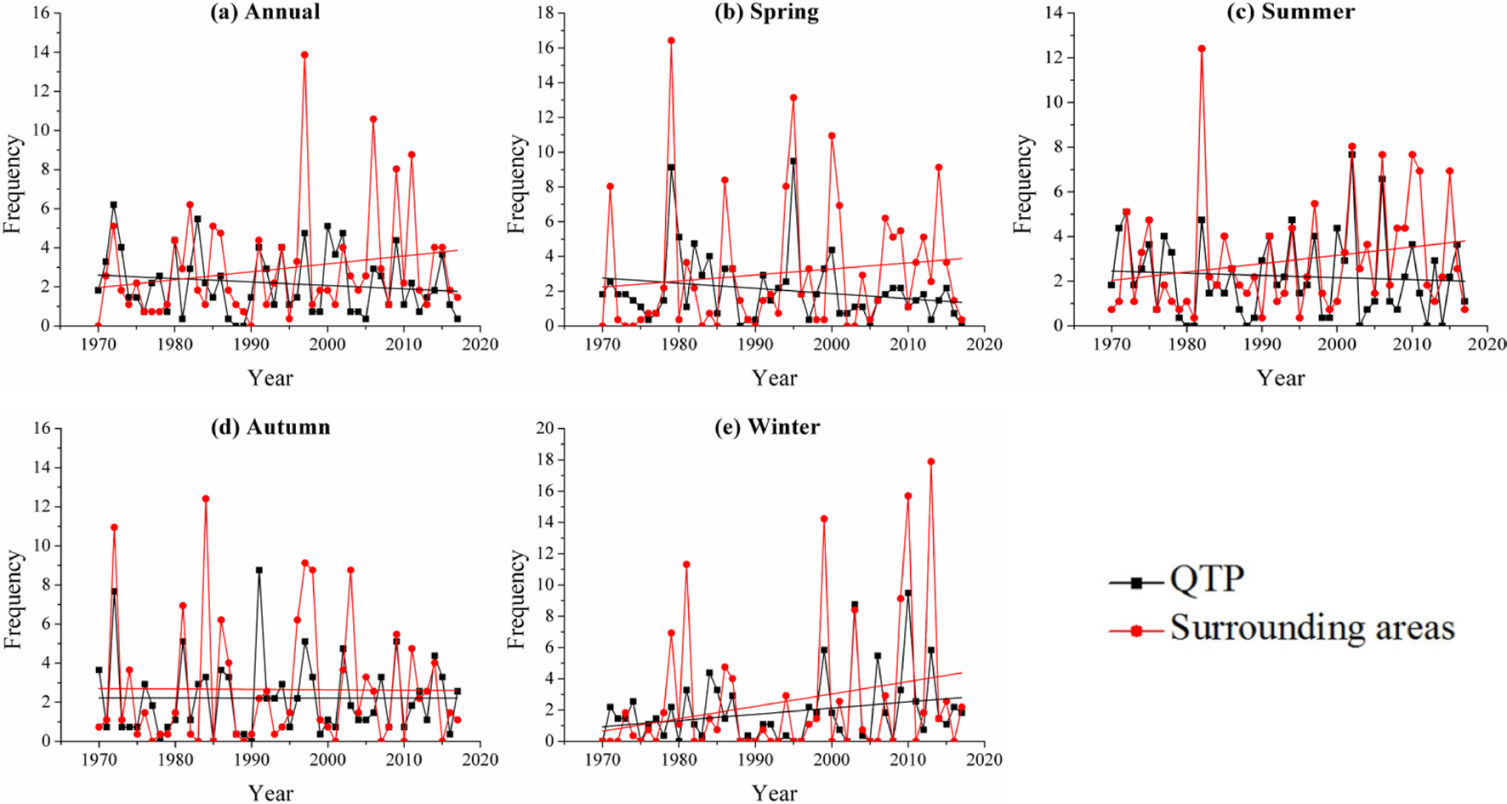
Annual and seasonal frequencies of severe drought: (**a**) Annual, (**b**) Spring, (**c**) Summer, (**d**) Autumn, (**e**) Winter in the QTP and its surrounding areas.

Figure 4 showed the annual and seasonal frequencies of severe drought events in the last half century. In general, the annual drought frequency of the QTP was decreasing while the surrounding areas exhibited an increasing trend. Before 1997, the differences of severe drought frequency between the QTP and its surrounding areas was not obvious (fluctuated in 0–6.2%), while in the last two decades, large differences have appeared with severe drought frequency in the surrounding areas increasing from 1.1 to 13.9% but that in the QTP decreasing from 0.4 to 5.1% (Fig. 4a). This indicated that the surrounding areas were more prone to severe drought in recent years. For the four seasons, the frequency of severe drought has decreased in spring, summer, but increased in winter in the QTP (Fig. 4e). In surrounding areas, however, spring, summer and winter experienced higher severe drought frequency increasing, and autumn had no obvious variations.

### Elevation dependence of SPEI trend

**Figure 5:**
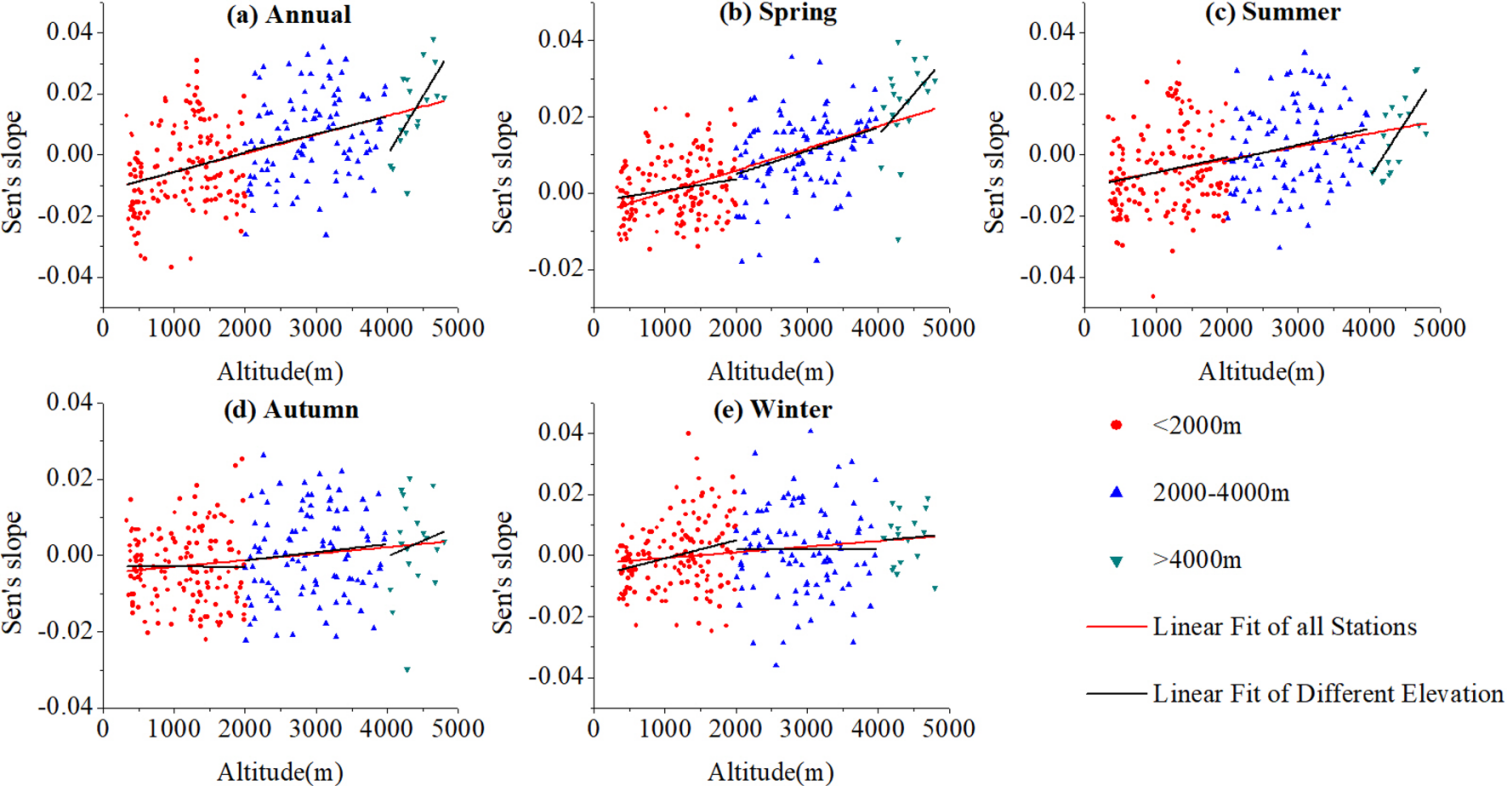
Relationships between SPEI trends and elevation: (**a**) Annual, (**b**) Spring, (**c**) Summer, (**d**) Autumn, (**e**) Winter in the QTP and its surrounding areas.

The relationship of Sen’s slope of SPEI series from 274 meteorological stations versus the elevation were analyzed in Fig. 5 and Table 3. The significance level adopted here was *p* < 0.05. Apparently, stations in the high-elevation ranges showed more rapidly increasing trends in SPEI series than those at lower elevations. For the entire study region, SPEI trends at different time scales increased with elevation and all passed the significance test, indicating a wetter trend in higher elevation. Specifically, it was positively correlated with elevation below 2000 m and passed the significance test except for autumn. Trends at elevation between 2000 m and 4,000 m were similar to that below 2000 m, and the annual, spring and summer passed the significance test. The most rapidly increasing trend occurred above 4,000 m and passed the significance test except for autumn and winter. Additionally, change magnitudes of SPEI trends with increasing elevation above 4,000 m were most robust in annual and summer (Fig. 5a–c), which were about 6.3 and 8.5 times that of the entire study region, respectively.

**Table 3:** The linear fit slopes of SPEI trends versus elevation on different altitudinal gradient.

Time	Study region	Below 2000 m	2000–4,000 m	Above 4,000 m	Above 4,000 m vs. Study region (multiple)
Annual	0.00614*	0.00627*	0.00582*	0.03875*	6.3
Spring	0.00577*	0.00295*	0.00608*	0.02159*	3.7
Summer	0.00424*	0.00498*	0.00527*	0.03607*	8.5
Autumn	0.00171*	− 0.000165	0.00212	0.00817	4.8
Winter	0.00184*	0.00593*	0.000119	0.00198	1.1

unit:/1,000 m. * indicates trends significant at *p* < 0.05.

In order to further explore the reasons, we analyzed the trends of three meteorological factors (temperature, precipitation and PET) that were used to compute SPEI. On the annual basis, for the elevation range above 4,000 m, the trends of temperature (T) and PET were negative but that of precipitation (P) was significantly positively correlated with elevation (Table 4), making a strong positive correlation between SPEI trend and elevation. For the other two elevation ranges (2000–4,000 m and below 2000 m), all trends of the three meteorological parameters were positively correlated with elevation, resulting in a weakly positive trend between of SPEI trend and elevation.

**Table 4:** Correlation coefficients of seasonal trends with elevation for T, P and PET in 1970–2017.

Seasons	Elevation	Correlation coefficient between the elevation and trends of meteorological factors		
		T	P	PET
Annual	< 2000	0.386*	0.336*	0.046
	2000–4,000	0.123	0.217*	0.002
	> 4,000	− 0.106	0.651*	− 0.126
Spring	< 2000	0.192*	0.139	− 0.047
	2000–4,000	− 0.069	0.218*	− 0.110
	> 4,000	− 0.036	0.246	− 0.132
Summer	< 2000	0.412*	0.350*	0.214*
	2000–4,000	0.159	0.161	0.021
	> 4,000	− 0.096	0.693*	− 0.339
Autumn	< 2000	0.349*	− 0.054	0.047
	2000–4,000	0.168	0.136	0.047
	> 4,000	− 0.012	0.064	− 0.018
Winter	< 2000	0.482*	0.118	0.014
	2000–4,000	0.150	− 0.087	0.132
	> 4,000	− 0.206	− 0.097	0.032

*indicates significant at *p* < 0.05.

At the seasonal scale, similar to that in annual, faster wetting trends were detected in spring and summer for the elevation above 4,000 m. In autumn, although T and PET trends were negative and P trend was positive with elevation, the correlation coefficients were smaller, resulting in unobvious wetting trends with elevation. The opposite changes of P and PET trends with elevation from other seasons led to the stable SPEI trend in winter. In general, the negative changes of T and PET trends and the positive change of P trend may contribute to the rapid wetter condition above 4,000 m. This could be better confirmed in annual, spring and summer, when the phenomenons were more obvious.

### Future persistence of drought

**Figure 6:**
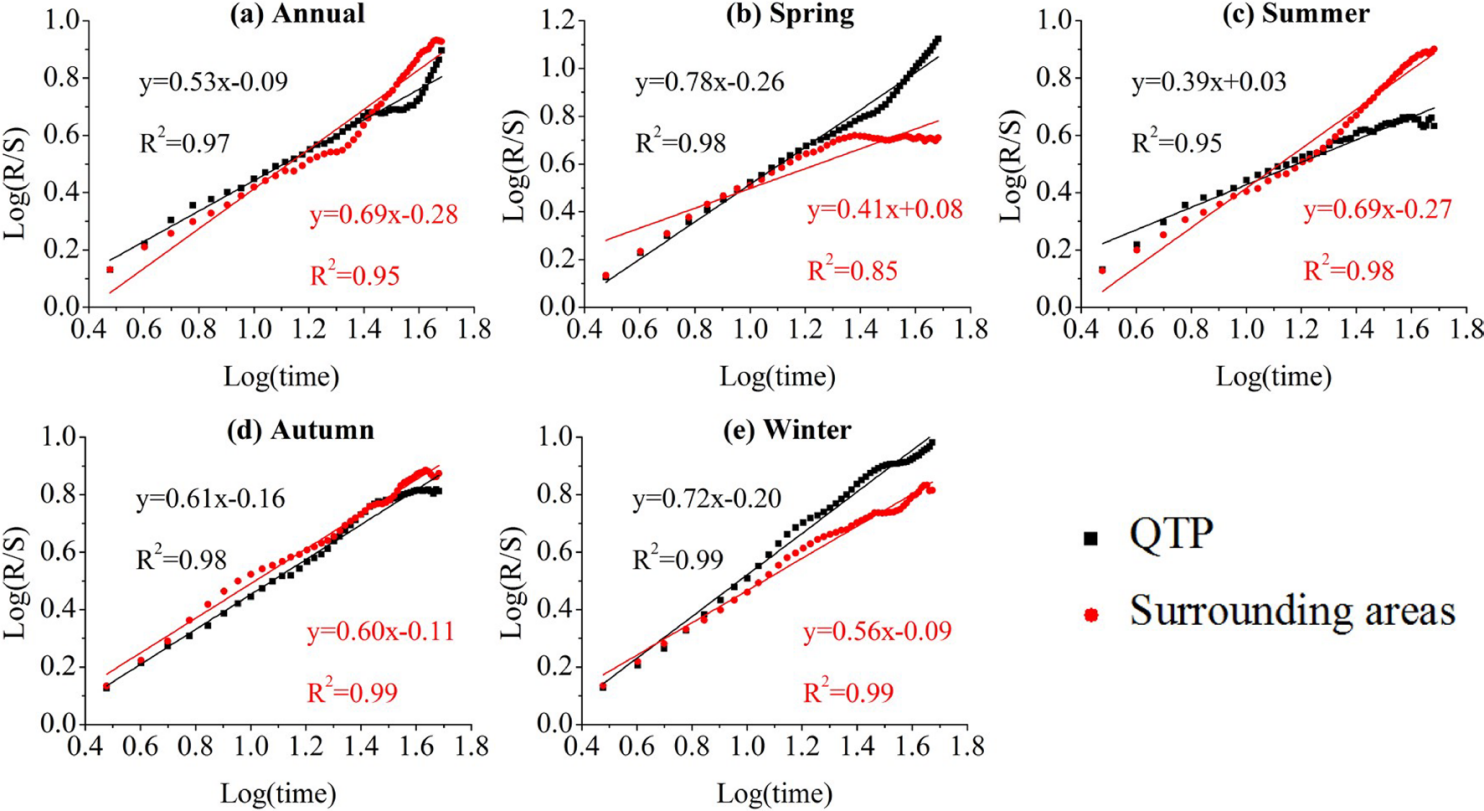
The rescaled range analysis of annual and seasonal SPEI series: (**a**) Annual, (**b**) Spring, (**c**) Summer, (**d**) Autumn, (**e**) Winter in the QTP and its surrounding areas.

According to the variation of SPEI in the QTP and its surrounding areas over the last half century, R/S analysis was performed to evaluate the long-term correlation of time series^42^. Figure 6 showed the results of R/S analysis in different seasons. In general, the Hurst index of annual SPEI in the QTP and surrounding areas were 0.53 and 0.69, respectively, indicating the drought may maintain current trends, i.e. the QTP got wetter and surrounding areas got drier in the future (Fig. 6a). The persistence of SPEI series in the study region showed clear seasonal differences. In the QTP, only summer (*H* = 0.39) exhibited small trends that were predicted to be drier in the future but other three seasons would likely maintain current trends (*H* were 0.78, 0.61 and 0.72, respectively), indicating a wetter climate. In the surrounding areas, the existing drying trend would continue in summer (*H* = 0.69) and autumn (*H* = 0.60), winter (*H* = 0.56) would keep the current wetter trend, and spring (*H* = 0.41) would reverse the current trend to drier climate. Relevant stakeholders should therefore pay attention to prevent the potential damage of drought events in summer.

## Discussion

### Rapid wetting trends at high-elevation regions

Elevation is of great importance in analyzing the climate spatial changes in mountainous regions, particularly in the QTP. This study explored the elevation dependence of drought as well as its possible causes in the QTP and the surrounding areas from 1970 to 2017. We showed that wetter trends were positively correlated with elevation (*p* < 0.05), with the change trend above 4,000 m being 6.3 times higher than below, and the most significant difference was as high as 8.5 times in summer. In terms of meteorological parameters, negative changes of temperature and PET trends and positive change of precipitation trend were detected above 4,000 m, particularly in annual, spring and summer. All of them have caused the rapid wetter condition at highland regions.

Together with global warming that glaciers in the QTP rapidly shrinks^43^, more streamflow was yielded in the region^25^. We concluded that the surrounding areas easily suffered from meteorological drought. However, this might be compensated by more streamflow at the low altitudes due to the upstream glacier melting. Glaciers are a uniquely drought resilient source of water, which may pose an important but underappreciated role in protecting downstream populations from the worst effects of droughts^44^. Glacial meltwater is also a source recharging river headwaters and downstream runoff in the QTP^45,46^. Previous studies showed that the runoff from headwater area of the QTP would be rising in future decades^47,48^, while the downstream precipitation was decreasing in the surrounding areas. This leads to the greater contribution and influence of runoff changes in the upstream on the downstream; for example, 70% runoff of Nujiang River in Yunnan Province comes from the upper reaches^49^. Affected by upstream water inflow, the scope and level of hydrological drought in downstream areas are weaker than those of meteorological drought^50,51^. Investigating the contribution of glaciers melting to the lower reaches of rivers is therefore necessary in the future, which can provide solid assistance for downstream drought adaptation.

It is worth noting that, although the QTP has become wetter, severe drought frequency in winter has increased, indicating that winter was more prone to severe droughts. This could not only reduce soil moisture, affect the growth of overwintering crops and the emergence of spring-sown crops but also endanger human and livestock drinking water. More violent fluctuations of severe drought events in the surrounding areas could be an omen of flash drought, which may bring devastating impacts on crop yields and water supply and further trouble the people as for less effective response^52–54^. The risk of flash drought in this region in the future thus deserved more attention of local authorities.

### Consistency of different drought indices

The selection of duration, indices and purposes, as well as the quality of data sources would all affect the results. Previously, many indices were used to identify the drought trends, frequency and severity in the QTP, such as the Palmer Drought Severity Index (PDSI)^55^, the Standardized Precipitation Index (SPI)^56^ and Temperature Vegetation Dryness Index (TVDI)^57^. Among them, PDSI may incorporate many climatic parameters (e.g. prior precipitation, moisture supply, runoff, evaporation demand) but is only applicable for mid- and long-term droughts due to the strong lagged autocorrelation^58,59^. SPI, on the other hand, only involves precipitation and shows high uncertainty in describing the drought in summer and winter; whereas SPEI simultaneously considers precipitation and evapotranspiration, and thus can accurately capture the effects of drought under the background of global warming.

To validate the findings of this study, we further compared with some previous achievements using other drought indices and data sources in the QTP (Table 5). Our results confirmed the wetting trend in the QTP with the most significant level in spring reported by previous studies^8,16,17,60^. Meanwhile, the use of different methods to calculated PET of the SPEI generated similar results, all indicating the eastern QTP becoming drier^14,17,32^. However, this spatial pattern showed conflict with Yu et al.^61^ and Wang et al.^18^ They reported a wetting trend in the eastern QTP and a significant drying trend in the north in spring and autumn. A number of stations, length of study period and slightly different regions may contribute to the differences, demonstrating the importance of adequate high-quality data and careful selection of study area when investigated the spatial–temporal changes of drought in the QTP.

**Table 5:** Comparisons with previous conclusions using different drought indices.

Region and station number	Period	Drought index	Main conclusions	References
QTP, 274	1970–2017	SPEI-TH	Got wetter mostly in spring Surrounding areas and several Eastern QTP stations became drier Increased severe drought in winter Drier summer in the future	This study
QTP, 83	1979–2011	P/PET	Significantly wetting in the northwestern QTP Insignificant drying in half of the eastern QTP	Gao et al.^8^
QTP, 74	1980–2014	SPEI-PM	Wetting in general Droughts detected in 75% stations Several northeastern and southern stations got drier	Liang et al.^14^
China, 541	1994–2013	SPI, SPEI-PM	Drought relieved in the QTP Significantly wetting in spring Weakly wetting in summer and autumn Insignificant drying in winter	Wang et al.^16^
China, 633	1961–2012	SPI, SPEI-PM	Wetting from the central to the northeastern part of the QTP Significant wetting in the middle/eastern QTP in April and May Drying trend of north QTP in February	Wang et al.^17^
Mainland of China except for the arid region	1960–2012	A 3-dimensional clustering method based on the SPI3, RDI3 and SPEI3	Significant wetting trend over the QTP	Xu et al.^32^
China, grid (0.5° × 0.5° resolution)	1961–2009	scPDSI	Significant wetting in the southern QTP Spring and autumn got drier in the northern QTP	Wang et al.^18^
China, 509	1951–2010	SPEI-TH	Wetting trend in the eastern QTP with the drought frequency decreased	Yu et al.^61^

## Conclusions

In this study, we analyzed the SPEI from 274 meteorological stations in the QTP and its surrounding areas during 1970–2017 and draw the following conclusions. Firstly, drought characteristics between the QTP and its surrounding areas have great differences: a wetting trend existed in the QTP with spring getting wet mostly at the rate of 0.0114/year, while the surrounding areas showed a drying trend, especially in Yunnan Province; the difference between SPEI in the QTP and its surrounding areas has increased from -0.19 in 1970 to 0.38 in 2017. Secondly, although a wetter climate in the QTP, we found the severe drought frequency in winter has substantiality increased, which indicated winter was more prone to severe drought. Thirdly, SPEI trend exhibited an elevation dependence, which generally increased with elevation, the change trend along elevation above 4,000 m was about 6.3 times higher than ones below 4,000 m. This was mainly caused by the decreasing temperature and PET trends and increasing precipitation trend. Lastly, in the future, the QTP and its surrounding areas would continue to be wetter and drier, respectively, and occurrence of future drought is most likely to increase in summer. The findings provided the basis for related researches, improved our understanding of the responses of dry and wet conditions to climate change across the QTP, and provided early warning for regional drought and references for drought disaster prevention and mitigation. However, the underlying mechanism of the drought pattern needs to be further explored.

## Data Availability

The meteorological datasets used in this study are available in the Data Center for Resources and Environmental Sciences, Chinese Academy of Sciences (https://www.resdc.cn/).
